# Corrigenda

**DOI:** 10.1107/S160053681201817X

**Published:** 2012-04-28

**Authors:** Haleden Chiririwa

**Affiliations:** a Department of Chemistry, University of Cape Town, Private Bag, Rondebosch 7707, South Africa

## Abstract

The affiliation of one of the authors and a source of funding are both added in the following papers: Chiririwa & Meijboom [*Acta Cryst.* (2011*a*), E**67**, m1496; *Acta Cryst.* (2011*b*), E**67**, m1497; *Acta Cryst.* (2011*c*), E**67**, m1498] and Chiririwa & Muller [*Acta Cryst.* (2012*a*), E**68**, m49; *Acta Cryst.* (2012*b*), E**68**, m116–m117].

## Abstract 2

Corrigenda for five articles.

Due to an oversight, an affiliation and a source of funding were omitted from five recent articles (Chiririwa & Meijboom, 2011*a* ▶,*b* ▶,*c* ▶; Chiririwa & Muller, 2012*a* ▶,*b* ▶). The affiliation of the correspondence author, Haleden Chiririwa, in all five articles should be ‘Department of Chemistry, University of Cape Town, Private Bag, Rondebosch 7707, South Africa’, as above. The University of Cape Town is also acknowledged for the use of their instrument. The acknowledgments section of the five papers should be appended with ‘This research was partially funded by Mintek and Project AuTEK’.
